# Summer Travelling

**Published:** 1856-08

**Authors:** 


### SUMMER TRAVELLING.

It is an almost universal practice for persons who travel, especially when children are along, to take a variety of cakes and sweetmeats. We earnestly warn our readers against the practice—it is in every way pernicious. Sweetmeats tempt the appetite, induce thirst, which when gratified produces a sensation of fulness and discomfort and crossness. It takes away the appetite of grown persons, clogs the stomach, and deranges the whole system.

There is nothing better for children and grown persons, than some crackers or cold bread, with some slices of ham. If really hungry, these will sustain nature, without being liable to the objections of sweetmeats. But for grown persons it is far best not to eat anything at all while travelling, except at regular meals. But if you are not sure of at least a full half hour, for actual sitting at the table, do not go to it. Take a sandwich, and travel on.

